# Joint effect of tobacco, alcohol, and oral HPV infection on head and neck cancer risk in the French West Indies

**DOI:** 10.1002/cam4.3327

**Published:** 2020-08-04

**Authors:** Aviane Auguste, Jacqueline Deloumeaux, Clarisse Joachim, Stanie Gaete, Leah Michineau, Cécile Herrmann‐Storck, Suzy Duflo, Danièle Luce

**Affiliations:** ^1^ Univ Rennes Inserm EHESP Irset (Institut de Recherche en Santé, Environnement et Travail) – UMR_S 1085 Pointe‐à‐Pitre France; ^2^ General Cancer Registry of Guadeloupe University Hospital of Guadeloupe Pointe‐à‐Pitre Guadeloupe; ^3^ Karubiotec™ Biological Resources Center Centre de Ressources Biologiques de Guadeloupe Pointe‐à‐Pitre Guadeloupe; ^4^ Martinique Cancer Registry UF 1441 Registre des Cancers Pôle de Cancérologie Hématologie Urologie Pathologie University Hospital of Martinique Fort‐de‐France Martinique; ^5^ Laboratory of Microbiology University Hospital of Guadeloupe Pointe‐à‐Pitre Guadeloupe; ^6^ Department of Oto‐Rhino‐Laryngology and Head and Neck Surgery University Hospital of Guadeloupe Pointe‐à‐Pitre Guadeloupe

**Keywords:** alcohol drinking, French West Indies, head and neck cancer, high‐risk oral HPV, tobacco smoking

## Abstract

We investigated the role of tobacco and alcohol consumption on the occurrence of head and neck squamous cell carcinomas (HNSCC), and the joint effects of these factors with oral human papillomavirus (HPV) infection in the French West Indies, in the Caribbean. We conducted a population‐based case‐control study (145 cases and 405 controls). We used logistic regression models to estimate adjusted odds ratios (OR) and their 95% confidence intervals (CI). Two‐way interactions were assessed on both multiplicative and additive scales. Current smoking (OR = 11.6, 95% CI = 6.7‐20.1), drinking more than five glasses of alcohol per day (OR = 2.7, 95% CI = 1.2‐4.7), and oral infection with High‐risk HPV (OR = 2.4, 95% CI = 1.1‐5.0) were significantly associated with HNSCC. The combined exposure to tobacco and alcohol produced a significant synergistic effect on the incidence of HNSCC. Oral infection with High‐risk HPV increased the risk of HNSCC in never smokers and nondrinkers. The effects of tobacco, alcohol, and of the combined exposure of tobacco and alcohol were substantially lower in HPV‐positive than in HPV‐negative HNSCC. This is the first case‐control study to investigate the role of tobacco smoking, alcohol drinking and oral HPV infection in an Afro‐Caribbean population. Although each of these risk factors has a significant effect, our findings indicate that tobacco and alcohol play a less important role in Hr‐HPV‐positive HNSCC. Further investigations are warranted notably on the interaction of these three risk factors by cancer site.

## Graphical abstract

This is the first case‐control study to investigate the role of tobacco smoking, alcohol drinking and oral human papillomavirus (HPV) infection in an Afro‐Caribbean population. We showed that these risk factors have significant independent effects on the occurrence of head and neck squamous cell carcinomas (HNSCC). A significant less than multiplicative interaction was found between alcohol drinking and high‐risk oral HPV infections. The concurrent exposure to tobacco and alcohol had a synergistic effect on HNSCC risk but only for high‐risk HPV‐negative cases.

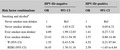

## 1 INTRODUCTION

Worldwide, more than 700 000 cases of head and neck cancer (including cancers of the oral cavity, pharynx, and larynx) are diagnosed each year.^1^ Tobacco smoking and alcohol drinking are the major risk factors for these cancers, their joint effect being at least multiplicative.^2^, ^3^ Human papillomavirus (HPV) is also a recognized cause of a subset of head and neck squamous cell carcinomas (HNSCC).^4^, ^5^ While the causal role of HPV16 in oropharyngeal cancer is well established, the role of other HPV genotypes or the association between HPV and other subsites of HNSCC is still debated.^6^ The manner in which tobacco, alcohol, and HPV interact on HNSCC risk remains unclear, with conflicting results. Some studies demonstrated a lack of association with tobacco and alcohol in HPV16‐positive HNSCC.^5^, ^7^, ^8^ Other more recent studies have shown that tobacco smoking and alcohol drinking have rather an independent role in the etiology of HPV16‐positive oropharyngeal cancer.^9^, ^10^, ^11^

Guadeloupe and Martinique are two French overseas territories in the French West Indies (FWI). The population is predominantly Afro‐Caribbean. Their incidence rates of head and neck cancer, especially in men, are among the highest in Latin America and the Caribbean,^1^ despite a relatively low prevalence of tobacco smoking and alcohol drinking.^12^ The prevalence of HPV in HNSCC and the distribution of HPV genotypes may vary substantially according to geographical regions^13^, ^14^, ^15^, ^16^ and ethnicity.^17^ Racial/ethnic differences in the effects of tobacco and alcohol on HNSCC have also been suggested.^18^

In order to elucidate the etiology of HNSCC in the FWI, we conducted a population‐based case‐control study. It was previously shown that oral infection with high‐risk HPV was associated with an increase in risk of HNSCC. Although oral infection with HPV16 was associated with oropharyngeal cancer, HPV16 was not the predominant genotype and there was a higher prevalence of other high‐risk HPV genotypes in cases than in controls.^19^

In this study, we aimed to investigate the role of tobacco and alcohol consumption on the occurrence of HNSCC, and the joint effects of these factors with oral HPV infection. To our knowledge, this is the first study on this topic in an Afro‐Caribbean population.

## 2 METHODS

### 2.1 Study population, data, and specimen collection

We extended a large population‐based case‐control study previously conducted in mainland France to the overseas French departments Guadeloupe and Martinique. The details of this study, the ICARE study, have been described elsewhere.^20^ A similar protocol and questionnaire were used for the study in the FWI, with some adaptations to the French West Indian context. Briefly, cases were identified in collaboration with the cancer registries. These registries have been in place since 1983 for Martinique and 2008 for Guadeloupe, and use standardized procedures for the recording of all cancer cases in each of these regions. Eligible cases were patients residing in the FWI, aged between 18‐75 years old, newly diagnosed with a primary, malignant, and histologically confirmed tumor of the oral cavity, pharynx, sinonasal cavities, and larynx between 1 April 2013 and 30 June 2016. Random digit dialing was used to select controls from the general population, using incidence density sampling. Controls were frequency matched to the cases by sex, age and region of residence (Guadeloupe and Martinique). Further stratification was applied to make the distribution by socioeconomic status of the control group similar to that of the general population.

Face‐to‐face interviews were conducted with cases and controls using a standardized questionnaire to ascertain sociodemographic characteristics and risk factors, in particular lifetime tobacco and alcohol consumption. During these interviews, Oragene^®^ OG‐500 kits (DNA Genotek) were used to collect a saliva sample from participants who gave their consent.

We initially identified 257 cases as potentially eligible, and 192 (74.7%) gave their consent to participate and were interviewed. After further review of clinical and pathological reports, 22 did not satisfy the inclusion criteria and were excluded from the study. Among the remaining 170 cases, 114 (72.3%) agreed to provide a saliva sample. Among the 497 eligible controls, 405 (81.5%) responded to the questionnaire and among them, 311 (76.2%) provided a saliva sample.

### 2.2 HPV detection and genotyping

Human papillomavirus‐integrated DNA from saliva samples was detected with the INNO‐LiPA^®^ kit, according to the manufacturer's instructions (INNO‐LiPA HPV Genotyping *Extra*; Innogenetics). The INNO‐LiPA HPV genotyping assay allows the detection of 32 HPV genotypes, which were classified as high‐risk HPV types (HPV16, HPV18, HPV31, HPV33, HPV35, HPV39, HPV45, HPV51, HPV52, HPV56, HPV58, HPV59, HPV68), probable high‐risk (HPV26, HPV53, HPV66, HPV70, HPV73, HPV82), low‐risk (HPV06, HPV11, HPV40, HPV42, HPV43, HPV44, HPV54, HPV61, HPV81), and other (HPV62, HPV67, HPV83, HPV89). The method for HPV detection has been described in detail elsewhere.^19^

### 2.3 Exposure variables

Detailed information on lifetime cigarette smoking history was recorded, for each period of identical smoking habits. The questionnaire included information on age at start and end of the period, number of cigarettes per day or per week, type of tobacco (blond vs black), filtered or not, inhalation pattern, and whether or not the product was manufactured or hand‐rolled. Ever cigarette smokers were defined as persons who smoked at least 100 cigarettes in their lifetime. Ex‐smokers were defined as persons who stopped smoking for at least 2 years. Smoking quantity was defined as the average number of cigarettes per day over the lifetime, and categorized into three groups (1‐10, 11‐20 and >20 cigarettes per day). Smoking duration was expressed in years and was divided into 4 categories (1‐20, 21‐30, 31‐40, >40 years). Never smoker was the reference category used for all smoking‐related variables in our analyses. Dichotomous variables (ever/never) were created for smoking pipes, cigars, chewing, and snuffing tobacco.

Lifetime alcohol drinking information was recorded as well, with for each period of regular consumption, the age at beginning and end, and the number of standard glasses per day, week or month for each type of alcoholic beverage (wine, beer, rum, and other strong spirits). For each type of beverage, ever daily alcohol drinking was defined as at least one glass per day during at least 1 year. The average number of glasses per day was calculated over the lifetime, regardless of the type of beverage, and categorized into three groups (<1, 1‐5, and >5 glasses per day). The reference category comprised subjects who never drank alcohol or who had drunk less than one glass per week. Alcohol duration was expressed in years and was divided into three categories (≤30, 31‐45, >45 years). Exposure to HPV was assessed in several manners. Subjects with at least one HPV infection of any type were classified as HPV‐positive, others were referred as HPV‐negative. The group of HPV‐positive was further divided in two categories: high‐risk‐HPV‐positive (at least one HPV type in the high‐risk group) and non‐high‐risk‐HPV‐positive. A final binary variable was used for the exposure to high‐risk‐HPV: high‐risk‐HPV‐positive vs high‐risk‐HPV‐negative, the latter category grouping HPV‐negative and non‐high‐risk‐HPV‐positive.

### 2.4 Statistical analysis

The current analysis was restricted to squamous cell carcinomas of the oral cavity (International Classification of Diseases 10th revision codes C00.3‐C00.9, C02.0‐C02.3, C03.0, C03.1, C03.9, C04.1, C04.8, C04.9, C05.0, C06.0‐C06.2, C06.8, and C06.9, n = 35), the oropharynx (ICD‐10 codes C01.9, C02.4, C05.1, C05.2, C09, C10, C 14.2, n = 58), the hypopharynx (ICD‐10 codes C12‐ C13, n = 19) and the larynx (ICD‐10 codes C32, n = 32). Our analysis included 145 cases and 405 controls. The association between smoking, alcohol and oral HPV infection and the occurrence of HNSCC was assessed by estimating odds ratios (ORs) adjusted for age, sex and recruitment site, and their 95% confidence intervals (CIs), using logistic regression models. The models for tobacco smoking were further adjusted for alcohol consumption. Models estimating the effect of alcohol were adjusted for smoking quantity and duration. Odds ratios associated with oral HPV were adjusted for smoking quantity, duration, and alcohol consumption. Two‐way interaction on a multiplicative scale was assessed by estimating Ψ, the multiplicative interaction parameter as follows, Ψ = OR_11_/(OR_01_*OR_10_). The 95% CI for Ψ was determined using the CI for the interaction term in the multivariate model. Two‐way interaction on an additive scale was assessed using the relative excess risk due to interaction (RERI), RERI = OR_11_ − OR_10_ − OR_01_ + 1. Asymptotic 95% CI were calculated for the RERI as described elsewhere.^21^ We also conducted analyses by cancer site (oropharynx/ non oropharynx). We grouped oral cavity, hypopharynx, and larynx because of sample size constraints.

Human papillomavirus status was missing for 147 (27%) subjects (53 cases and 94 controls) who refused to provide a saliva sample, and for three controls for whom the quality of the specimen was considered inadequate for HPV detection. In addition, missing data were observed for smoking status (one case) smoking quantity (19 cases, 3 controls), smoking duration (6 cases, 1 control), alcohol quantity (4 controls), and alcohol duration (27 cases 56 controls). We used multiple imputations by chained equations to deal with missing data.^22^ The imputation model contained all the basic characteristics of the study subjects (age, sex recruitment site and education level), variables related to alcohol and smoking (ever daily alcohol drinking, quantity of alcohol, smoking status, smoking duration, and smoking quantity), HPV status (low‐risk, probable high‐risk, high‐risk, and other HPV types) and the case‐control status. All variables in the imputation model which had missing values were imputed for our analyses. We generated 20 datasets. We also performed a complete case analysis, on a dataset containing only observed data. Results were similar to those from the imputed datasets, despite wider confidence intervals (see Supporting Information). Statistical analysis was performed using SAS 9.4 software (SAS Institute).

## 3 RESULTS

### 3.1 Characteristics of study population

Table 1 shows socio‐demographic characteristics of HNSCC cases and controls. The majority of subjects in our study were between 55 and 64 years old and were men. A little under half of the cases had only primary school education (42.8%) compared to 23.2% of the controls.

**TABLE 1:** Socio‐demographic characteristics of HNSCC cases and controls

Characteristics	Cases		Controls	
	n = 145	col%	n = 405	col%
Age (y)				
<45	3	2.1	62	15.3
45‐54	40	27.6	107	26.4
55‐64	61	42.1	129	31.9
>65	41	28.3	107	26.4
Sex				
Women	18	12.4	99	24.4
Men	127	87.6	306	75.6
Recruitment site				
Guadeloupe	95	65.5	245	60.5
Martinique	50	34.5	160	39.5
Education level				
Primary school	62	42.8	94	23.2
Secondary school	51	35.2	161	39.8
High school diploma	17	11.7	53	13.1
Tertiary education	15	10.3	97	24.0

Note

French West Indies, 2013‐2016.

Abbreviation: HNSCC, head and neck squamous cell carcinomas.

### 3.2 Tobacco, alcohol, oral HPV, and HNSCC risk

Table 2 shows multivariate ORs of HNSCC and 95% CI associated with tobacco smoking, alcohol drinking and oral HPV. Current smokers were significantly 11 times more likely to develop HNSCC compared to never smokers. Ex‐smokers were only twice as likely to develop a HNSCC compared to never smokers. The risk increased with the quantity and duration of tobacco smoking. Significant increases in risk by more than 10‐fold were observed for more than 20 cigarettes per day, and for more than 30 years. We studied as well the combination between smoking quantity and duration. We observed that duration had a greater role in HNSCC etiology than the quantity. Persons who smoked for shorter periods of time (less than 30 years) had a lower risk for developing HNSCC regardless of the quantity of cigarettes smoked per day.

**TABLE 2:** Multivariate OR of HNSCC and 95% CI associated with tobacco smoking, alcohol drinking, and oral HPV infection

Risk factor	Cases		Controls		OR	95% CI
	n = 145	col%	n = 405	col%		
Tobacco smoking^a^						
Never smoker	30	21.8	263	64.9	1	Ref
Smoking status						
Current smoker	88	61.1	52	12.8	11.59	6.69‐20.08
Former smoker	26	18.1	90	22.2	2.28	1.24‐4.17
Missing	1		0			
Quantity (cigarette per day)						
1‐10	35	27.8	71	17.7	4.17	2.33‐7.46
11‐20	35	27.8	51	12.7	6.11	3.39‐11.04
>20	26	20.6	17	4.2	10.69	4.89‐23.41
Missing	19		3			
Duration (y)						
1‐20	9	6.5	57	14.1	1.43	0.64‐3.23
21‐30	17	12.2	37	9.2	3.94	1.92‐8.07
31‐40	42	30.2	23	5.7	12.25	6.16‐24.37
>40	41	29.5	24	5.9	13.28	6.61‐26.68
Missing	6		1			
≤20 cigarettes per day						
During ≤ 30 y	16	12.8	84	21.0	2.00	1.05‐3.81
During > 30 y	53	42.4	37	9.2	12.19	6.68‐22.24
>20 cigarettes per day						
During ≤ 30 y	6	4.8	7	1.8	7.10	2.13‐23.70
During > 30 y	20	16.0	10	2.5	15.38	6.03‐39.19
Missing	20		4			
Alcohol quantity^b^ (glasses per day)						
Never or occasionally	51	35.2	216	53.9	1	ref
<1	8	5.5	73	18.2	0.40	0.17‐0.93
1‐5	45	31.0	84	21.0	1.24	0.70‐2.20
>5	41	28.3	28	7.0	2.36	1.18‐4.73
Missing	0		4			
Alcohol duration^b^ (y)						
≤30	25	17.2	129	31.9	1	ref
31‐45	67	46.2	161	39.8	1.69	0.88‐3.23
>45	26	17.9	59	14.8	3.56	1.45‐8.74
Missing	27		56			
Type of beverage^b^ (daily drinking)						
Wine	38	26.2	54	13.3	1.35	0.76‐2.39
Beer	34	23.5	35	8.6	1.83	0.98‐3.42
Rum	75	51.7	61	15.1	2.90	1.74‐4.84
Other strong spirits	13	9.0	12	3.0	1.82	0.68‐4.89
Oral HPV status^c^						
HPV‐negative	60	65.2	228	73.9		ref
Any HPV	32	34.8	80	26.1	1.44	0.82‐2.53
HPV‐Non‐high risk	13	14.1	50	16.3	0.80	0.35‐1.83
HPV‐High risk	19	20.7	30	9.8	2.37	1.13‐4.97
Missing	53		97			

Note

French West Indies, 2013‐2016.

Abbreviations: CI, confidence interval; HNSCC, head and neck squamous cell carcinomas; HPV, human papillomavirus; OR, odds ratio.

^a^ Model adjusted for age, sex, recruitment site, alcohol consumption (glasses per day).

^b^ Model adjusted for age, sex, recruitment site, tobacco smoking as the combination of quantity (cigarettes per day) and duration (y).

^c^ Model adjusted for age, sex, recruitment site, tobacco smoking status, alcohol consumption (glasses per day).

Compared to never smokers, the risk of HNSCC was slightly greater for the persons who smoked only black tobacco than blond tobacco alone (OR = 5.97, 95% CI = 2.80‐12.73; OR = 4.67, 95% CI = 2.55‐8.54, respectively) (data not shown). The ORs were higher for those who inhaled deeply cigarette fumes (OR = 5.20, 95% CI = 2.94‐9.18) than for those who never inhaled (OR = 3.76, 95% CI = 1.57‐8.96) or inhaled a little (OR = 3.53, 95% CI = 1.85‐6.75) (data not shown). Cigarettes without filters (2.5%), hand‐rolled cigarettes (2.8%), pipe (5.0%), and cigars (3.6%) were uncommon in our study population and were not associated with the risk of HNSCC (data not shown). It should be noted that all cigar smokers and all pipe smokers but one case had also smoked cigarettes. No subject had chewed tobacco and only one case had snuffed.

We observed a significant inverse association for those persons who drank less than one glass per day in relation to HNSCC, when compared to nondrinkers. We found that drinking more than five glasses of alcohol per day increases the risk of HNSCC by two‐fold. We observed as well an increase, although not significant, in HNSCC risk for persons who drank between 1 and 5 glasses per day. Compared to persons who drank alcohol for 30 years or less, the risk of HNSCC increased significantly among persons who drank for more than 45 years.

Rum was the most frequently consumed alcoholic beverage in our study population regardless of case‐control status. The daily consumption of rum and beer increased the risk significantly by two fold compared to the persons who never drank rum or beer daily. In contrast, daily consumption of wine and other strong spirits did not increase the risk significantly compared to nondaily drinkers.

In terms of oral HPV infections, no significant association with HNSCC was found for persons tested positive for HPV when compared to HPV‐negative subjects. Non‐high‐risk HPV types as well did not show any significant difference in risk to HPV‐negative subjects. On the other hand, subjects positive for Hr‐HPV types were twice as likely to develop HNSCC compared to Hr‐HPV‐negative subjects. Among Hr‐HPV types, we detected HPV16 in 4 cases and 2 controls. HPV18 was found only in 2 cases and 1 control. The other Hr‐HPV types which were detected in greater proportion among cases than controls were HPV33 (3 cases 1 control), HPV51 (3 cases 0 controls), HPV52 (8 cases 12 controls), and HPV56 (5 cases 6 controls).

We analyzed tobacco, alcohol, and oral HPV risk among oropharyngeal and non‐oropharyngeal subsites separately; these results did not change in terms of direction of the association observed in the analyses with all HNSCC cases (data not shown).

### 3.3 Joint effect of risk factors and HNSCC risk

Table 3 shows the multivariate ORs, their 95% CIs, and measures of two‐way interaction for combined exposures to risk factors, for HNSCC and by subsite. Compared to never smokers and nondrinkers, never smokers who drank alcohol daily had a nonsignificant increase in HNSCC risk (OR = 2.01, 95% CI = 0.87‐4.61) whereas smokers who did not drink alcohol were 3 times more likely to have HNSCC (OR = 3.57, 95% CI = 1.89‐6.74). The joint effect of tobacco and alcohol was more than multiplicative but not significant for HNSCC (Ψ = 2.01, 95% CI = 0.75‐5.37). However, a significant interaction was observed on the additive scale for tobacco and alcohol (RERI = 9.82, 95% CI = 3.06 to 16.57). Never smokers positive for Hr‐HPV were significantly more likely to have HNSCC when compared with Hr‐HPV‐negative never smokers (OR = 4.74, 95% CI = 1.45‐15.50). Moreover, Hr‐HPV‐negative ever smokers had an even greater risk of HNSCC (OR = 6.30, 95% CI = 3.44‐11.52). Negative interactions, although not significant, between Hr‐HPV and smoking were observed on both the multiplicative (Ψ = 0.30, 95% CI = 0.07‐1.24) and the additive scale (RERI = −1.07, 95% CI = −8.28 to 6.15). Hr‐HPV‐positive nondrinkers and Hr‐HPV‐negative drinkers were both significantly more likely to have HNSCC than Hr‐HPV‐negative nondrinkers. The joint effect of alcohol and Hr‐HPV on HNSCC risk was less than additive (RERI = −3.30, 95% CI = −8.01 to 1.42) and significantly less than multiplicative (Ψ = 0.24, 95% CI = 0.06‐0.99). Negative interactions involving oral Hr‐HPV were consistently more marked for alcohol than tobacco. We also performed the above interaction analyses on oropharyngeal and non‐oropharyngeal squamous cell carcinomas separately. Although the difference in effect size and trends did not differ significantly between subsites, the effect of all the studied risk factors appeared to be of greater magnitude for the oropharynx than the non‐oropharynx cases. In particular, oral Hr‐HPV in never smokers and in nondrinkers was found to be significant for only oropharyngeal cancer.

**TABLE 3:** Multivariate OR, their 95% CI, and measures of two‐way interactions of combined exposures between risk factors for HNSCC and by subsite

Risk factor combinations	All cases	Oropharynx	Non‐Oropharynx
	OR (95% CI)	OR (95% CI)	OR (95% CI)
Smoking and alcohol^a^			
Never smoker‐non drinker	1 (ref)	1 (ref)	1 (ref)
Never smoker‐drinker	2.01 (0.87‐4.61)	2.81 (0.75‐10.48)	1.79 (0.64‐5.01)
Ever smoker‐non drinker	3.57 (1.89‐6.74)	5.59 (2.06‐15.20)	2.75 (1.24‐6.11)
Ever smoker‐drinker	14.39 (8.02‐25.82)	19.37 (7.56‐49.61)	13.23 (6.62‐26.45)
Ψ (95% CI)	2.01 (0.75‐5.37)	1.23 (0.28‐5.67)	2.69 (0.80‐9.11)
RERI (95% CI)	9.82 (3.06‐16.57)	11.96 (−1.32 to 25.24)	9.69 (2.25‐17.13)
Smoking and Hr‐HPV^b^			
Never smoker‐Hr‐HPV−	1 (ref)	1 (ref)	1 (ref)
Never smoker‐Hr‐HPV+	4.74 (1.45‐15.50)	5.23 (1.10‐24.86)	3.26 (0.64‐16.60)
Ever smoker‐Hr‐HPV−	6.30 (3.44‐11.52)	8.82 (3.47‐22.38)	5.11 (2.46‐10.61)
Ever smoker‐Hr‐HPV+	8.98 (3.85‐20.94)	10.09 (2.94‐34.56)	7.53 (2.77‐20.43)
Ψ (95% CI)	0.30 (0.07‐1.24)	0.22 (0.03‐1.38)	0.45 (0.07‐2.94)
RERI (95% CI)	−1.07 (−8.28 to 6.15)	−2.97 (−14.35 to 8.42)	0.16 (−6.67 to 6.99)
Alcohol and Hr‐HPV^c^			
Non drinker‐Hr‐HPV−	1 (ref)	1 (ref)	1 (ref)
Non drinker‐Hr‐HPV+	4.43 (1.50‐13.11)	4.76 (1.31‐17.32)	3.39 (0.69‐16.78)
Drinker‐Hr‐HPV−	3.20 (1.76‐5.82)	3.85 (1.65‐8.97)	3.06 (1.47‐6.39)
Drinker‐Hr‐HPV+	3.33 (1.27‐8.73)	2.40 (0.62‐9.30)	3.70 (1.24‐11.09)
Ψ (95% CI)	0.24 (0.06‐0.99)	0.13 (0.02‐0.80)	0.36 (0.05‐2.40)
RERI (95% CI)	−3.30 (−8.01 to 1.42)	−5.21 (−11.93 to 1.52)	−1.75 (−6.93 to 3.43)

Note

French West Indies, 2013‐2016.

Abbreviations: ; CI, confidence interval; HNSCC, head and neck squamous cell carcinomas; HPV, human papillomavirus; OR, odds ratio; Phi (Ψ), measure of interaction on a multiplicative scale (interaction term); RERI, relative excess risk due to interaction.

^a^ Model adjusted for age, sex, recruitment site.

^b^ Model adjusted for age, sex, recruitment site and ever daily alcohol consumption.

^c^ Model adjusted for age, sex, recruitment site, tobacco smoking as the combination of quantity (cigarettes per day) and duration (y).

Table 4 shows the associations for combined exposures to tobacco smoking and alcohol drinking, stratified by Hr‐HPV status. In the Hr‐HPV‐negative subgroup, the trend was similar to the effect sizes and the measures of interaction for all study participants together. The Hr‐HPV‐positive subgroup on the other hand had overall lower effect sizes for the tobacco‐alcohol profiles compared to their Hr‐HPV‐negative counterparts.

**TABLE 4:** Multivariate OR, their 95% CI, and measures of interaction of combined two‐way exposures between tobacco smoking and alcohol drinking for HNSCC stratified by Hr‐HPV status

Risk factor combinations	HPV‐Hr‐negative		HPV‐Hr‐positive	
	OR	95% CI	OR	95% CI
Smoking and alcohol^a^				
Never smoker‐non drinker	1	Ref	1	Ref
Never smoker drinker	3.09	1.03‐9.22	0.56	0.05‐6.72
Ever smoker‐non drinker	4.89	1.99‐12.03	1.41	0.27‐7.32
Ever smoker‐drinker	23.43	10.11‐54.30	3.57	0.88‐14.48
Ψ (95% CI)	1.55	0.43‐5.58	4.52	0.21‐97.84
RERI (95% CI)	16.45	1.76‐31.16	2.59	−1.65 to 6.84

Note

French West Indies, 2013‐2016.

Abbreviations: CI, confidence interval; HNSCC, head and neck squamous cell carcinomas; HPV, human papillomavirus; OR, odds ratio; RERI, relative excess risk due to interaction; Ψ, multiplicative interaction parameter.

^a^ Model adjusted for age, sex, recruitment site.

## 4 DISCUSSION

Our findings provide new insight into the role of tobacco, alcohol, and oral HPV infection and their combined effects on the occurrence of HNSCC in the FWI.

Similarly to other studies, we found that the risk of HNSCC increased with the duration and intensity of smoking, and the duration had a greater effect than the average number of cigarettes per day.^3^, ^23^ Rum was found to be the beverage which conferred the greatest risk of HNSCC compared to other alcoholic beverages. This observed association for rum is likely to result from it being the most frequently consumed alcoholic beverage in the FWI rather than an independent effect of the alcohol concentration.^24^ The inverse association we found for light alcohol drinking (<1 glass per day) was consistent with a French study^25^; however, a recent meta‐analysis reported pooled estimates that suggested rather a nonsignificant positive association between light alcohol drinking and head and neck cancer.^26^ The increased risk we observed for the long period of alcohol consumption was also supported by a study from the INHANCE consortium.^23^ Although not significant we observed a more than multiplicative effect of the combined of exposure to tobacco and alcohol on HNSCC risk which was of similar magnitude to a study conducted within the INHANCE Consortium.^2^ The few studies assessing the additive interaction for tobacco and alcohol conducted their analysis in individual HNSCC subsites and reported super‐additive interactions of varying degrees.^27^, ^28^, ^29^

Oral Hr‐HPV infections were significantly associated with HNSCC, regardless of Hr‐HPV genotype. A study conducted in Canada did not find any significant association with HNSCC and Hr‐HPV types excluding HPV16.^10^ In our study, only four cases and two controls were positive for HPV16, and the effect of Hr‐HPV on HNSCC was maintained after removing HPV16‐positive subjects. These results could be suggestive of a greater role of non‐HPV16 high risk types in HNSCC carcinogenesis in the FWI compared to other populations, as also suggested for cervical infections.^30^

Concerning the joint effect of tobacco and HPV, and alcohol and HPV, we found some evidence of a negative interaction on both the additive and multiplicative scale. In particular, the combined effect of alcohol and HPV was significantly less than multiplicative contrary to previous studies which revealed more synergistic relationships.^11^, ^31^, ^32^ Despite the negative interactions that we found, tobacco and alcohol still increased the risk of HNSCC regardless of HPV status. The effect of tobacco, alcohol and of the combined exposure to tobacco and alcohol were however substantially lower in HPV‐positive than in HPV‐negative HNSCC, which is indicative of a more pre‐dominant role of tobacco and alcohol in HPV‐negative HNSCC as described in previous studies which investigated HPV16 specifically.^5^, ^7^, ^8^

We studied the prevalence of oral HPV infection and we are not able to distinguish between recent and long‐term infections, which complicates the interpretation of the results on the interactions. Smoking has been shown to increase the likelihood of acquisition^33^ and persistence^34^, ^35^, ^36^ of oral HPV infection in some studies, whereas other studies found no association between smoking and oral HPV incidence^34^, ^37^, ^38^, ^39^ or persistence of oral HPV infection.^40^, ^41^ It has been suggested that smoking‐induced secretory leukocyte protease inhibitor expression may prevent HPV infections.^42^, ^43^ Additional studies are needed to fully elucidate the complex role of smoking in oral HPV natural history. Alcohol intake was not found to be associated with incidence^33^, ^34^, ^37^, ^38^ or persistence.^34^, ^40^, ^41^

Analyses by subsite did not reveal important differences with regards to the effects of tobacco and alcohol; although point estimates were higher in oropharyngeal cancer than in non‐oropharyngeal cancer, the confidence intervals were wide and the effect of traditional risk factors was similar in both subsites, as previously shown.^9^

Our data on the joint effect of tobacco, alcohol and Hr‐HPV on the occurrence of oropharyngeal cancer were supported by previous reports.^9^, ^10^ We found that Hr‐HPV was associated with a significant increase in risk of oropharyngeal cancer in never smokers and in nondrinkers. These significant associations were not present in the non‐oropharyngeal cases. In addition, the measures of interaction for the joint exposure with each of the risk factors and oral Hr‐HPV‐positive infections were more marked in the oropharyngeal cases than the non‐oropharyngeal cases. Furthermore, the significant sub‐multiplicative interaction between alcohol and Hr‐HPV was observed exclusively in the oropharynx. These observations support an etiological role of oral Hr‐HPV specific to oropharyngeal cancer, as in previous studies.^7^, ^8^, ^31^, ^32^ We demonstrated that alcohol alone did not play a role in Hr‐HPV‐positive oropharyngeal cancer as described previously.^9^, ^10^ Our results did not provide strong evidence for a role of tobacco in oropharyngeal carcinogenesis regardless of HPV status, contrarily to a recent study which emphasized the existence of a positive association in HPV16‐related oropharyngeal cancer.^9^

Our study presents some limitations. The statistical power was low to detect interactions. In addition, the small sample size limited the detail in our analyses. In particular, we were not able to assess three‐way interactions by subsite as we would have liked with tobacco, alcohol and Hr‐HPV. We had 27% missing data for HPV in our sample. To handle missing data, we used a multiple imputation procedure that has been shown to result in less biased and more precise estimates than the exclusion of individuals with missing data.^22^ The case‐control design coupled with the lack of temporal sequence in HPV data made it difficult to put forward a more precise mechanism between the risk factors and HPV in HNSCC risk. We had very few subjects infected with HPV16, which made comparisons with other studies difficult.^7^, ^8^, ^9^, ^10^ Furthermore, the use of oral HPV detection to assess the HPV status may have resulted in misclassification, which is however likely to be nondifferential. Oral HPV detection has been shown to have good specificity but moderate sensitivity for HPV‐positive HNSCC tumors.^44^ Despite the limitations imposed by oral HPV detection, this method is indicative of the site of infections compared to HPV serology which is not site‐specific.

Considering the participation of the local cancer registries in the selection of cases and the methods employed to select the controls, we believe that no major selection bias has occurred in this study. Cases included in our study had a distribution by age, sex and cancer site similar to that of the cases recorded in the cancer registries; therefore, our sample can be considered representative of the HNSCC cases. The selection method for the controls was previously shown to produce unbiased samples, thus our controls could be regarded as being representative of persons of similar age and sex from the general population.^20^ We confirmed the representativeness of the tobacco and alcohol distribution in our control group to FWI population after comparison with the data from a national health survey.^12^

## 5 CONCLUSION

This is the first case‐control study to investigate the role of tobacco smoking, alcohol drinking and oral HPV infection in an Afro‐Caribbean population. We showed that these risk factors have significant independent effects on the occurrence of HNSCC. Overall, our findings suggest a less important role of tobacco and alcohol in Hr‐HPV‐positive HNSCC. The precise mechanisms driving these interactions on HNSCC risk are yet to be elucidated and further investigations are warranted notably on the interaction of these three risk factors simultaneously.

## CONFLICT OF INTEREST

The authors have no conflict of interest.

## AUTHORS' CONTRIBUTIONS

JD, CJ, SG, SD, and DL participated in the study concept and design, and collected the data. AA, LM, and DL conducted the quality control of data. AA, SG, CH, and DL participated in the interpretation of data. AA and DL performed cleaning of final dataset, statistical analysis, and prepared the manuscript draft. All authors participated in manuscript editing, review, and approved the final version.

## ETHICAL APPROVAL

French Data Protection Authority (CNIL, Commission Nationale de l’Informatique et des Libertés) no. DR‐2015‐2027; IRB INSERM no. 01‐036.

## Supporting information

Tables S1‐S3

## Data Availability

The data that support the findings of this study are available from the corresponding author upon reasonable request.
