# Supplementary material for: Joint effect of tobacco, alcohol, and oral HPV infection on head and neck cancer risk in the French West Indies
Source: Cancer Med. 2020 Aug 4;9(18):6854–63. doi: 10.1002/cam4.3327 (PMC7520253; doi:10.1002/cam4.3327)
Supplement: Supplementary file 1 — Tables S1‐S3 [file CAM4-9-6854-s001.pdf]

Supplementary Materials

Multivariate logistic regression analyses performed on observed data

Supplementary table 1: Multivariate OR of HNSCC and 95%CI associated with tobacco smoking, alcohol drinking and oral (observed data).

Risk factor	Cases n=145	col%	Controls n=405	col%	OR	95%CI
**Tobacco smoking**[a]						
Never smoker	30	(21.8)	263	(64.9)	1	ref
**Smoking status**						
Current smoker	88	(61.1)	52	(12.8)	9.04	(5.13-15.92)
Former smoker	26	(18.1)	90	(22.2)	1.97	(1.05-3.68)
Missing	1		0			
**Quantity (cigarette/day)**						
1 to 10	35	(27.8)	71	(17.7)	3.63	(2.01-6.57)
11 to 20	35	(27.8)	51	(12.7)	4.71	(2.56-11.04)
>20	26	(20.6)	17	(4.2)	7.79	(3.58-16.95)
Missing	19		3			
**Duration (years)**						
1 to 20	9	(6.5)	57	(14.1)	1.06	(0.43-2.59)
21 to 30	17	(12.2)	37	(9.2)	3.19	(1.51-6.76)
31 to 40	42	(30.2)	23	(5.7)	8.89	(4.39-18.04)
> 40	41	(29.5)	24	(5.9)	10.84	(5.33-22.05)
Missing	6		1			

**≤ 20 cigarettes/day**						
during ≤ 30 years	16	(12.8)	84	(21.0)	1.56	(0.79-3.09)
during >30 years	53	(42.4)	37	(9.2)	9.76	(5.29-18.02)
**> 20 cigarettes/day**						
during ≤ 30 years	6	(4.8)	7	(1.8)	6.03	(1.80-20.23)
during > 30 years	20	(16.0)	10	(2.5)	10.27	(4.10-25.71)
Missing	20		4			
**Alcohol quantity (glasses/day)**						
Never or occasionally	51	(35.2)	216	(53.9)	1	ref
<1 glass/day or <7 glasses/week	8	(5.5)	73	(18.2)	0.50	(0.21-1.19)
1 to 5 glasses/day	45	(31.0)	84	(21.0)	1.61	(0.88-2.96)
>5 glasses/days	41	(28.3)	28	(7.0)	2.50	(1.18-5.29)
Missing	0		4			
**Alcohol duration[b] (years)**						
≤ 30	25	(17.2)	129	(31.9)	1	ref
31-45	67	(46.2)	161	(39.8)	1.75	(0.87-3.45)
> 45	26	(17.9)	59	(14.8)	4.51	(1.77-11.45)
Missing	27		56			
**Type of beverage[b] (daily drinking)**						
Wine	38	(26.2)	54	(13.3)	1.43	(0.78-2.64)
Beer	34	(23.5)	35	(8.6)	2.02	(1.06-3.83)
Rum	75	(51.7)	61	(15.1)	3.01	(1.76-5.17)
Other strong spirits	13	(9.0)	12	(3.0)	1.73	(0.64-4.70)
**Oral HPV Status[c]**						
HPV-Negative	60	(65.2)	228	(73.9)		ref
Any HPV	32	(34.8)	80	(26.1)	1.23	(0.66-2.30)

HPV-Non-high risk	13	(14.1)	50	(16.3)	0.74	(0.33-1.70)
HPV-High risk	19	(20.7)	30	(9.8)	2.10	(0.95-4.88)
Missing	53		97			

French West Indies, 2013-2016

a: Model adjusted for age, sex, recruitment site, alcohol consumption (glasses/day)

b: Model adjusted for age, sex, recruitment site, Tobacco smoking as the combination of quantity (cigarettes/day) and duration (years)

c: Model adjusted for age, sex, recruitment site, Tobacco smoking status, alcohol consumption (glasses/day)

Supplementary table 2: Multivariate OR, their 95% CI, and measures of two-way interactions between risk factors for HNSCC, and by subsite (observed data).

Risk factor combinations	All cases		Oropharynx		Non-Oropharynx	
	OR	(95%CI)	OR	(95%CI)	OR	(95%CI)
**Smoking and Alcohol**[a]						
Never Smoker-Non Drinker	1	(ref)	1	(ref)	1	(ref)
Never Smoker-Drinker	1.97	(0.86-4.52)	2.69	(0.72-10.05)	1.79	(0.64-5.01)
Ever smoker-Non drinker	3.57	(1.89-6.74)	5.59	(2.06-15.21)	2.75	(1.24-6.11)
Ever Smoker-Drinker	14.31	(7.97-25.68)	19.13	(7.46-49.08)	13.23	(6.62-26.45)
Ψ (95%CI)	2.04	(0.76-5.45)	1.27	(0.29-5.66)	2.69	(0.80-9.11)
RERI (95% CI)	9.77	(3.06 to 16.49)	11.85	(-1.29 to 25.00)	9.69	(2.25 to 17.13)
**Smoking and Hr-HPV**[b]						
Never Smoker-Hr-HPV-	1	(ref)	1	(ref)	1	(ref)
Never Smoker-Hr-HPV+	7.81	(2.35-25.88)	9.86	(1.86-52.36)	3.30	(0.60-18.03)
Ever smoker-Hr-HPV-	6.67	(3.29-13.49)	9.66	(3.19-29.23)	5.22	(2.22-12.26)
Ever Smoker-Hr-HPV+	6.70	(2.39-18.76)	7.91	(1.76-35.64)	5.53	(1.66-18.46)
Ψ (95%CI)	0.13	(0.03-0.57)	0.08	(0.01-0.64)	0.32	(0.05-2.52)
RERI (95% CI)	-6.78	(-17.86 to 4.30)	-10.61	(-30.61 to 9.40)	-1.99	(-9.88 to 5.91)
**Alcohol and Hr-HPV**[c]						
Non Drinker-Hr-HPV-	1	(ref)	1	(ref)	1	(ref)
Non Drinker-Hr-HPV+	6.54	(2.12-20.15)	7.83	(1.73-35.38)	3.03	(0.55-16.68)
Drinker-Hr-HPV-	5.32	(2.60-10.91)	7.92	(2.79-22.52)	4.30	(1.78-10.41)
Drinker-Hr-HPV+	4.18	(1.38-12.61)	4.95	(1.01-24.40)	3.40	(0.96-12.10)
Ψ (95%CI)	0.12	(0.03-0.56)	0.08	(0.01-0.60)	0.26	(0.05-2.62)
RERI (95% CI)	-6.68	(-15.65 to 2.28)	-9.80	(-25.66 to 4.94)	-2.93	(-9.89 to 4.04)

French West Indies, 2013-2016

a: Model adjusted for age, sex, recruitment site

b: Model adjusted for age, sex, recruitment site and ever daily alcohol consumption

c: Model adjusted for age, sex, recruitment site, Tobacco smoking as the combination of quantity (cigarettes/day) and duration (years)
Ψ: Phi, measure of interaction on a multiplicative scale (interaction term)
RERI: Relative Excess Risk due to Interaction

Supplementary table 3: Multivariate OR, their 95% CI, and measures of two-way interaction of combined exposures to tobacco smoking and alcohol drinking for HNSCC stratified by Hr-HPV status (observed data).

Risk factor combinations	HPV-Hr-Negative		HPV-Hr-Positive	
	OR	95% CI	OR	95% CI
**Smoking and Alcohol**[a]				
Never Smoker –Non Drinker	1	(ref)	1	(ref)
Never Smoker -Drinker	6.71	(1.95-23.09)	0.40	(0.03-4.83)
Ever smoker- Non drinker	6.49	(2.14-19.75)	0.40	(0.05-3.22)
Ever Smoker- Drinker	43.66	(15.43-123.54)	1.94	(0.44-8.57)
Ψ (95%CI)	1.00	(0.23-4.28)	12.25	(0.47-319.23)
RERI (95% CI)	31.46	(10.09-52.83)	2.14	(1.23-3.06)

French West Indies, 2013-2016

a: Model adjusted for age, sex, recruitment site

Ψ: multiplicative interaction parameter

RERI: Relative Excess Risk due to Interaction
